# The Clonal Expansion Dynamics of the HIV-1 Reservoir: Mechanisms of Integration Site-Dependent Proliferation and HIV-1 Persistence

**DOI:** 10.3390/v13091858

**Published:** 2021-09-17

**Authors:** Yang-Hui Jimmy Yeh, Kerui Yang, Anya Razmi, Ya-Chi Ho

**Affiliations:** Department of Microbial Pathogenesis, Yale University School of Medicine, New Haven, CT 06519, USA; yang-hui.yeh@yale.edu (Y.-H.J.Y.); kerui.yang@yale.edu (K.Y.); anya.razmi@yale.edu (A.R.)

**Keywords:** HIV-1 latent reservoir, HIV-1 cure, clonal expansion, HIV-1 insertional mutagenesis, HIV-1 integration site-dependent proliferation, aberrant HIV-1 RNA splicing, persistent nonsuppressible low-level viremia, HIV-1 proviral landscape, immune selection pressure, HIV-1 suppressing agents

## Abstract

More than 50% of the HIV-1 latent reservoir is maintained by clonal expansion. The clonally expanded HIV-1-infected cells can contribute to persistent nonsuppressible low-level viremia and viral rebound. HIV-1 integration site and proviral genome landscape profiling reveals the clonal expansion dynamics of HIV-1-infected cells. In individuals under long-term suppressive antiretroviral therapy (ART), HIV-1 integration sites are enriched in specific locations in certain cancer-related genes in the same orientation as the host transcription unit. Single-cell transcriptome analysis revealed that HIV-1 drives aberrant cancer-related gene expression through HIV-1-to-host RNA splicing. Furthermore, the HIV-1 promoter dominates over the host gene promoter and drives high levels of cancer-related gene expression. When HIV-1 integrates into cancer-related genes and causes gain of function of oncogenes or loss of function of tumor suppressor genes, HIV-1 insertional mutagenesis drives the proliferation of HIV-1-infected cells and may cause cancer in rare cases. HIV-1-driven aberrant cancer-related gene expression at the integration site can be suppressed by CRISPR-mediated inhibition of the HIV-1 promoter or by HIV-1 suppressing agents. Given that ART does not suppress HIV-1 promoter activity, therapeutic agents that suppress HIV-1 transcription and halt the clonal expansion of HIV-1-infected cells should be explored to block the clonal expansion of the HIV-1 latent reservoir.

## 1. Introduction

Antiretroviral therapy (ART) effectively blocks new rounds of HIV-1 infection and suppresses HIV-1 plasma viral load to clinically undetectable levels. However, as early as three days after infection, millions of HIV-1-infected cells establish the HIV-1 reservoir in peripheral blood [1,2,3] and tissues, particularly lymphoid tissues such as lymph nodes and gut-associated lymphoid tissues [4,5]. Despite decades of suppressive ART, the latent reservoir [1,2,3] persists lifelong [6,7]. Whenever an HIV-1-infected individual stops ART, viral rebound is inevitable. Unlike antibacterial agents that kill susceptible bacteria, ART blocks HIV-1 enzyme function or viral entry but does not kill HIV-1-infected cells; in fact, ART is not cytotoxic. Presumably, infected cells should be recognized and eliminated by the host innate and adaptive immune system [8,9,10,11]. However, under effective ART, HIV-1 evades innate and adaptive host immune clearance. For innate immune responses, cytosolic viral RNA should induce recognition by cytosolic RIG-I-like receptors. However, once HIV-1 integrates into the human genome, HIV-1 RNA transcribed from integrated proviruses appears similar to cellular mRNA and thus does not induce innate sensing. For adaptive immune responses, HIV-1-infected cells evade immune clearance by cytotoxic CD8^+^ T lymphocytes (CTLs) through different mechanisms, such as rapid development of CTL escape mutations [12,13,14], inducing CTL exhaustion [15,16,17], downregulating MHC I-mediated antigen presentation [18], and using defective HIV-1-infected cells as decoys to distract CTLs from killing the latent reservoir [19]. Therefore, the HIV-1 latent reservoir persists despite long-term suppressive ART.

## 2. The Clonally Expanding HIV-1-Infected Cells Are the Major Barrier to Cure

The half-life of the HIV-1 latent reservoir is estimated to be 44 months [6], despite the improvement of ART into more potent and less toxic regimens [7]. The persistence of HIV-1-infected cells is a dynamic process: they increase by clonal expansion and decrease by immune clearance through cytotoxic T lymphocyte and natural killer cell killing. More than 50% of the latent reservoir is maintained through clonal expansion [20,21,22], and the frequency of clonally expanded cells increases over time [23]. These clonally expanding HIV-1-infected cells are the major barrier to a cure.

HIV-1 infects cells that have CD4 receptor and CCR5 or CXCR4 coreceptors, such as CD4^+^ T cells, macrophages, and dendritic cells. HIV-1 infection of CD4^+^ T cells significantly helps the life-long persistence of the virus. This is because HIV-1 hides in memory CD4^+^ T cells that (a) undergo clonal expansion upon antigen stimulation [24,25,26,27], (b) dynamically switch from an activated state to a quiescent and transcriptionally inactive latent phase, and (c) are maintained through homeostatic proliferation [28]. In normal immune responses, naïve CD4^+^ T cells become activated following cognate antigen stimulation, differentiate into memory T cells, and proliferate from one cell into many cells (creating a T cell clone). This clonal expansion of memory CD4^+^ T cells is a normal immune response to antigen stimulation. Of note, memory CD4^+^ T cells undergo homeostatic proliferation faster than naïve CD4^+^ T cells [29]. The estimated proliferation rate is highest in effector memory CD4^+^ T cells (0.042/day) [29,30], followed by central memory CD4^+^ T cells (0.01/day) [29,30] and naïve CD4^+^ T cells (0.004/day) [29]. For example, HIV-1-infected cells are more clonal in the effector memory CD4^+^ T cells and are not clonal in the naïve CD4^+^ T cells [31]. T cell memory resides in long-lived clones of T cells, not in long-lived individual cells. This preferential proliferation of memory T cells confers an advantage to HIV-1 when integrated into memory T cells.

Once the antigen is removed, memory CD4^+^ T cells will return to a quiescent memory state that is transcriptionally inactive. Upon cognate antigen stimulation, these resting memory CD4^+^ T cells become activated and proliferate into a T cell clone again. Although many cells die of viral cytotoxic effects during productive HIV-1 infection, a substantial number of HIV-1-infected cells survive and follow the clonal expansion and contraction dynamics of CD4^+^ T cells. For example, upon antigen stimulation, one HIV-1-infected cell can proliferate into many infected cells; upon cognate antigen removal, HIV-1-infected CD4^+^ T cells return to the quiescent memory state. These transcriptionally inactive cells do not have active transcription factors such as NF-κB and NFAT in the nucleus and therefore do not induce effective HIV-1 transcription. By residing in these transcriptionally inactive resting CD4^+^ T cells, HIV-1 does not make viral antigens and therefore cannot be recognized by the immune system. Furthermore, these HIV-1-infected memory CD4^+^ T cells undergo homeostatic replenishment through cytokines such as interleukin (IL)-7 [28]. These homeostatic cytokines induce T cell homeostatic proliferation without inducing HIV-1 reactivation [32,33]. Therefore, during homeostatic proliferation, HIV-1-infected cells proliferate but do not express antigens that can be recognized by the immune system. Upon the next round of antigen stimulation, the HIV-1-infected memory CD4^+^ T cells are activated and proliferate into a T cell clone. Antigen stimulation induces T cell activation through NF-κB and NFAT signaling. Because the HIV-1 LTR promoter has NF-κB and NFAT binding sites, antigen stimulation will presumably also activate HIV-1 and reverse latency. However, antigen activation appears to be stochastic: T cell activation levels follow a gradient, not an all-or-none on-off switch. This means that antigen stimulation, which should presumably reactivate all HIV-1 and expose the infected cells to immune clearance, can only activate a subset of HIV-1-infected cells. Therefore, HIV-1-infected cells persist over time by residing in CD4^+^ T cells, following the normal T cell expansion and replenishment responses, and hiding in the quiescent state that cannot be fully reactivated despite T cell activation.

## 3. HIV-1 Expression from Clonally Expanded HIV-1-Infected Cells Causes Nonsuppressible and Persistent Low-Level Viremia of Predominant HIV-1 Plasma Clones

**Figure 1:**
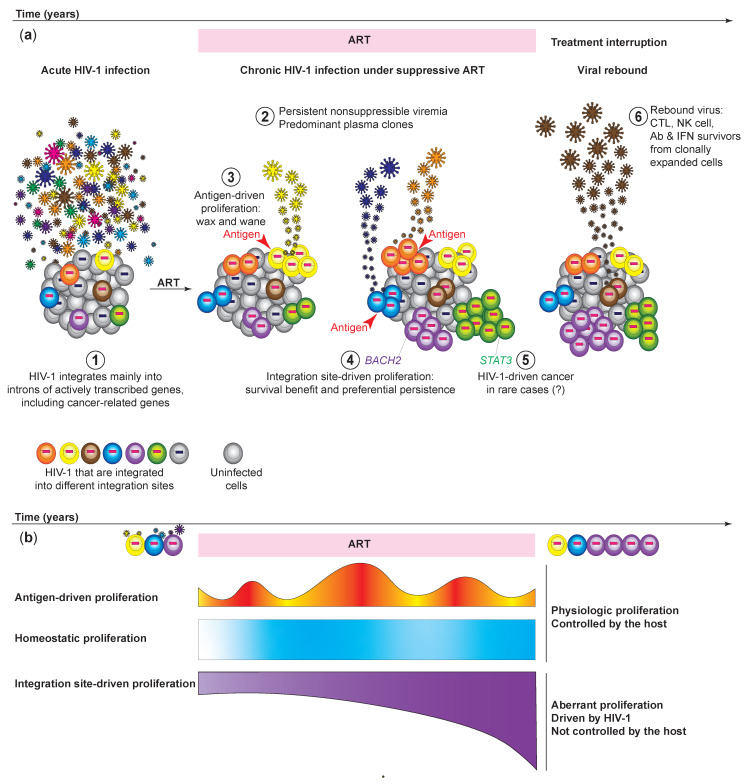
The clonal expansion dynamics of HIV-1-infected cells. (**a**) Clonally expanded cells as a source of persistent nonsuppressible low-level viremia and viral rebound. (1) HIV-1 integration into specific locations of cancer-related genes is present during acute infection but not yet enriched. (2) Persistent nonsuppressible low-level viremia of predominant plasma clones originate from clonally expanded cells. (3) Antigen stimulation causes clonal expansion of CD4^+^ T cells regardless of integration sites, but these clones wax and wane. (4) HIV-1 integration into specific locations of cancer-related genes persistently drives the proliferation of HIV-1-infected cells. (5) In rare cases, HIV-1 integration into cancer-related genes may cause cancer in the infected cells. (6) Clonally expanding HIV-1-infected cells can contribute to viral rebound. (**b**) Unlike antigen-driven proliferation and homeostatic proliferation, which are under host control, HIV-1 insertional mutagenesis may induce uncontrolled proliferation of infected cells.

Despite effective ART and drug adherence, HIV-1-infected individuals can still have intermittent low-level of viremia (or blips) [34,35] (Figure 1a). HIV-1 viral sequencing analysis by the Siliciano group revealed that the plasma viruses during low-level viremia are dominated by a few clones of HIV-1, known as the predominant plasma clones [36]. These predominant plasma clones wax and wane over time [33]. In a case report by Simonetti et al., an HIV-1-infected individual developed nonsuppressible viremia of one predominant plasma clone [25]. This virus was sensitive to the ART regimen but continued to produce high levels of viremia despite ART. HIV-1 sequence analysis revealed that this predominant plasma clone actually originated from a CD4^+^ T cell clone that expanded in response to the squamous cell carcinoma in this HIV-1-infected individual [25]. The predominant plasma clone decreased in number when the cancer was under control and increased when the cancer progressed, suggesting that it is the tumor antigen response that drives the T cell proliferation and HIV-1 expression [25]. With technology advancements to track HIV-1 sequences and integration site simultaneously in higher throughput, the Mellors group identified the plasma viral sequences in nonsuppressible viremia from more HIV-1-infected individuals [37]. They identified the origin of these viral clones by identifying identical HIV-1 sequences between the viruses in the plasma and the proviruses in the CD4^+^ T cells in the blood. By tracking the integration sites of these HIV-1 proviruses, Halvas et al. found that the nonsuppressible persistent low-level of viremia and the predominant plasma clones actually originate from large T cell clones in the peripheral blood [37]. Of note, the HIV-1 integration sites were not in cancer-related genes in these clonal expansion events, suggesting that antigen stimulation alone is sufficient to drive the proliferation of the infected cells regardless of integration site. Furthermore, near-full length proviral sequencing in these case reports found that the proviruses in these clonally expanded HIV-1-infected cells are likely replication competent. It remains unclear why these HIV-1-infected cells do not die of viral cytopathic effects upon latency reversal. Single-cell transcriptome analysis of clonally expanded HIV-1-infected cells suggested that some infected cells may upregulate cellular factors that promote the survival of the infected cells [38,39], but whether these factors mechanistically promote the survival of the infected cells remains to be validated. Altogether, these results resolved the long-term question of the source of nonsuppressible and persistent low-level viremia: the clonally expanding HIV-1-infected cells undergo stochastic activation (presumably by antigen stimulation), leading to intermittent HIV-1 expression and virion release into the plasma. Since activation is based on stochastic antigen stimulation, these predominant plasma clones wax and wane intermittently. While ART blocks new rounds of infection from these virions, ART does not block HIV-1 expression or clonal expansion of the infected cells, and thus low-level viremia is not suppressible by ART and persists over time. These findings reveal the need for new therapeutic strategies to halt the ongoing proliferation of HIV-1-infected cells and the chronic antigen presentation from these infected cells.

## 4. Clonally Expanded HIV-1-Infected Cells Serve as a Source of Viral Rebound after Treatment Interruptions

The absence of an immediate viral rebound after treatment interruption indicates long-term ART-free remission and a clinically significant reduction in the HIV-1 latent reservoir. Thus, identifying the source of viral rebound after treatment interruptions is a top priority in HIV cure research. The finding that clonally expanded cells are a source for persistent low-level viremia [25,37] under ART suggests that clonally expanded cells can likely be a source of viral rebound. Blood sampling before and after analytical treatment interruptions (ATIs) in therapeutic intervention clinical trials may provide more clues regarding the origin of viral rebound. The Chomont group developed the simultaneous T cell receptor sequence (TCR), integration site, and provirus sequencing (STIP-Seq) method that captures HIV-1 clonal expansion dynamics [40]. Briefly, STIP-seq involves sorting HIV-1 p24^+^ single-cells for genomic DNA extraction and phi29-mediated whole-genome amplification. The amplified DNA is then split into separate aliquots for TCR sequencing, integration site sequencing, and HIV-1 near full-length proviral genome sequencing. Some HIV-1 proviruses in clonally expanded CD4^+^ T cells captured during viral suppression are identical to plasma viruses captured during ATIs, suggesting that clonally expanded HIV-1-infected CD4^+^ T cells can contribute to viral rebound [40] (Figure 1a).

In some cases, phylogenetic analysis of HIV-1 sequences before and after ATI suggests that the rebound virus after ATI does not always match the sequences of the HIV-1 latent reservoir before ATI [41,42]. Additional studies revealed the reasons why the latent reservoir (captured by limiting dilution viral outgrowth cultures) and the rebound virus do not always match. First, since blood sampling typically only takes ~100 mL to 500 mL out of ~5 L peripheral blood (0.2–1% of total blood volume), matching sequences between viral rebound and the latent reservoir may not be found in all studies due to under-sampling. Second, upon latency reversal, cells harboring HIV-1 proviruses can be killed by CTLs and NK cells in vivo, but this immune selection pressure is not measured in standard viral outgrowth cultures (in which CD4^+^ T cells are cultured without CTLs and NK cells). Third, HIV-1 viruses that caused rebound need to survive in vivo immune selection pressures that do not exist in ex vivo viral outgrowth cultures, such as autologous immunoglobulins G (IgGs) [43] and type I interferon responses [44]. Fourth, HIV-1-infected cells may hide in anatomical sanctuaries (such as the central nervous system) [45] or immune sanctuaries (such as B cell follicles in the lymph node) [46] that may cause viral rebound but are not captured by peripheral blood sampling [46]. For example, HIV-1 sequence and integration site analysis identified the same sequences between the blood and lymph node compartments in some studies [47,48] but not all [49], indicating that blood sampling can reflect HIV-1 reservoir in both blood and lymphoid compartments in some cases; however, under-sampling of the heterogeneous HIV-1 reservoir remains an issue. Nevertheless, these results suggest that HIV-1 proviruses in the clonally expanded HIV-1-infected cells need to be resistant to both type I interferon responses and antibodies in the plasma for a successful exponential outgrowth and viral rebound. Blood samples from HIV-1-infected individuals remain the most accessible clinical samples for long-term follow-ups, while HIV-1-infected cells in tissues may encounter a different immune selection microenvironment. Therefore, mechanisms of HIV-1 persistence in tissues require further investigation.

## 5. Near Full-Length HIV-1 Proviral Genome Profiling Distinguishes Intact versus Defective HIV-1 Proviruses and Identifies Clonally Expanded HIV-1-Infected Cells

### 5.1. HIV-1 Proviral Sequencing Provides an Indirect Method for Identifying Clonal Expansion of the Infected Cells

Because of the high error rate of HIV-1 reverse transcriptase [4.1 × 10^−3^ (~1/244) [50] to 5.9 × 10^−4^ (~1/1700)(error per number of base pairs) [51]], two proviruses having the exact same proviral sequence indicate that the two proviruses come from clonally expanded HIV-1-infected cells, as opposed to independent infection events from the same virus. However, the HIV-1 proviral sequence was challenging to capture: only ~1000 per million CD4^+^ T cells (~0.1%) have HIV-1-infected cells [52]. Bulk HIV-1 DNA sequencing mixes different HIV-1 genomes and cannot capture individual HIV-1 proviral sequences. Ho et al. developed a near full-length HIV-1 proviral genome profiling method in 2013 [53]: briefly, DNA from clinical samples is diluted into 96 well plates for near full-length nested HIV-1 PCR, capturing ~9 kb of the HIV-1 proviral genome. Under limiting dilution, meaning that <20% of the 96 well plate wells are positive for HIV-1 PCR signal, Poisson statistics indicates that each of these HIV-1 PCR positive wells contains only one HIV-1 provirus (single proviral genome), as opposed to a mixture of gene fragments from different proviruses (with >90% confidence). This method allows us to examine the HIV-1 proviral landscape. In particular, only <12% of HIV-1 proviruses are intact; the remaining ~90% of the HIV-1 proviruses are defective due to large internal deletions (45.5%), APOBEC3G-mediated hypermutations (32.4%), packaging signal and major splice donor defects (6.5%), and point mutations (3.8%) [53]. Of note, only cells harboring intact HIV-1 proviruses are defined as the HIV-1 latent reservoir; cells harboring defective HIV-1 proviruses are HIV-1-infected cells but do not produce infectious virions and are not the latent reservoir. In this study, reconstructed intact proviruses demonstrated replication fitness comparable to NL4-3 references, suggesting that intact HIV-1 near-full length proviral sequences confer replication competence [53]. Capturing the same HIV-1 near full-length proviral genomes from different cells identifies clonal expansion of HIV-1-infected cells [54]. Follow-up studies characterizing HIV-1 near-full length proviral genomes using next-generation sequencing [as Full-Length Individual Provirus Sequencing (FLIPS) by the Sarah Palmer group [55] and full-length HIV-1 sequencing assay by the Lichterfeld Group] [56] captured clonal expansion of HIV-1-infected cells at higher throughput. Overall, these near-full length proviral sequencing methods enable capturing the clonal expansion of intact versus defective HIV-1 proviruses, providing HIV-1 full-length genome information that integration site methods cannot offer.

### 5.2. The Highly Diverse HIV-1 Pol and Env Sequences from Limiting Dilution Viral Outgrowth Culture Serve as Proxies for Measuring Clonally Expanded HIV-1-Infected Cells

While near full-length proviral sequencing provides the genome landscape of both intact and defective HIV-1 proviruses, this labor- and resource-intensive method captures very few intact HIV-1 proviruses, given that the majority (>90%) of HIV-1 proviruses are defective [53,54,57]. Consequently, after sequencing > 100 HIV-1 full-length proviruses researchers are only able to capture < 10 HIV-1 intact proviruses. Methods that can enrich the capture efficiency of cells harboring intact HIV-1 proviruses can better visualize the clonal expansion dynamics of the latent reservoir. By sequencing highly diverse HIV-1 regions in *env* (C2-V4 [22] or gp160) [21] or *gag-pol* (*p6-PR-RT*) [20] of positive wells from limiting dilution viral outgrowth cultures (suggesting that the supernatant from each positive well contains only one virus) and mapping out their phylogenetic trees, three independent studies found that >50% of the latent reservoir undergo the clonal expansion. These studies identified understanding the clonal expansion of the latent reservoir as a top priority in HIV-1 cure research.

## 6. Integration Site Analysis Provides a Definite Proof of the Clonal Expansion of Infected Cells

The chance of two HIV-1 integration events into the same chromosome location among the three billion base pairs of the human genome is extremely unlikely. Therefore, cells harboring HIV-1 proviruses that are integrated into the same nucleotide in the human chromosome come from clonal expansion of the same infected cell, not two independent infections. Many integration site analysis methods have been developed over the years, including enzymatic fragmentation followed by inverse PCR [58,59,60] and sonication fragmentation followed by ligation-mediated PCR (LM-PCR) [61]. Yet it was not until 2014, when large scale integration site analysis was performed on HIV-1 clinical samples, that researchers appreciated integration site-dependent clonal expansion.

### 6.1. Lessons from HTLV-1: High-Throughput Integration Site Analysis Captures the Clonal Expansion Dynamics of Infected Cells

Earlier, in 2011, the Bangham group first found that human T lymphotropic virus type 1 (HTLV-1)-infected cells undergo clonal expansion [61]. This discovery was made by an elegant method that captures almost 100,000 HTLV-1 integration sites using blood samples from HTLV-1-infected individuals. HTLV-1-infected cells with the same integration site are “clones”. They arise from clonal expansion (or proliferation) from the same cell. Although researchers were able to capture HTLV-1 integration sites, it was challenging to distinguish whether detection of many copies of the same integration sites came from one integration site that was PCR amplified in vitro or from many different HTLV-1-infected cells that had clonally expanded. To solve this problem, the Bangham group sheared the DNA from blood samples by sonication. This shearing creates unique breakpoints of DNA fragments. After adaptor ligation and HTLV-1-specific PCR amplification, both the HTLV-1 integration site and the unique breakpoint are captured. Therefore, using the unique breakpoints as barcodes, HTLV-1 integration sites from different cells will have unique breakpoints. By counting the number of HTLV-1 integration sites with different breakpoints, the number of clonally expanded HTLV-1-infected cells can be accurately measured [61]. This method was later termed “sonic abundance” by the Bushman group in 2012 [62].

### 6.2. The Discovery of Clonally Expanded HIV-1-Infected Cells by Mapping HIV-1 Integration Sites

**Figure 2:**
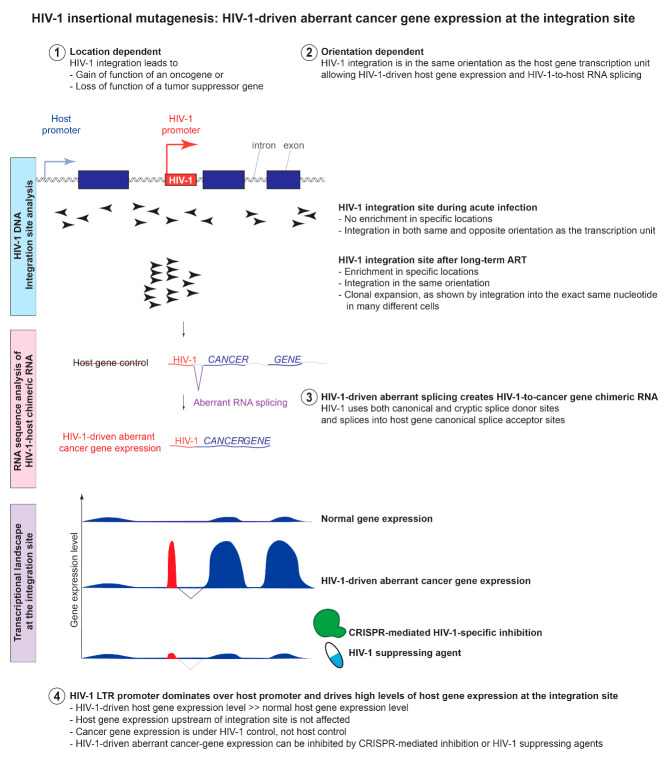
Mechanisms of HIV-1 insertional mutagenesis and HIV-1 integration site-dependent proliferation.

Although HIV-1 integration site analysis captures the clonal expansion dynamics of HIV-1-infected cells, both methods profiling HIV-1 integration sites can only capture a small proportion of the HIV-1 genome and therefore cannot distinguish intact HIV-1 proviruses from defective proviruses. Several HIV researchers developed different methods to capture clonally expanded HIV-1-infected cells. In 2014, two back-to-back studies at Science by Maldarelli et al. [63] and Wagner et al. [23] captured hundreds to thousands of HIV-1 integration sites. The Maldarelli study applied the sonic abundance method to HIV-1, while the Wagner study developed a novel integration site loop amplification (ISLA) to capture the unique HIV-1 integration sites. Among their findings were that up to 43% of HIV-1-infected cells are clonally expanded [63], and clonally expanded cells can persist for more than 11 years [63] and increase over time [23] (Figure 1b). Of note, there is an enrichment of HIV-1 integration into cancer-related genes [23], such as *BACH2*, *MKL2*, and *STAT5B* [23,63]. For example, while cancer-related genes constitute 5.19% of the human genome, 12.5% of the HIV-1 integration sites are in cancer-related genes [23]. Strikingly, integration sites observed in vivo are distinct from those observed during in vitro infections: both studies found that HIV-1 integration is enriched in two cancer-related genes *BACH2* and *MKL2* in introns near the translation start site, and in the same orientation as the transcription unit [23]. In contrast, HIV-1 infection in vitro shows integration sites throughout the *BACH2* and *MKL2* genes, with no enrichment of integration located near the translation start site and no enrichment of integration in the same orientation [63] (Figure 2). These breakthrough studies shifted the paradigm that the expansion of HIV-1-infected cells arises from new rounds of infection; instead, despite effective ART, HIV-1-infected cells proliferate and increase over time. Overall, studying HIV-1 integration site and clonal expansion dynamics provides critical insights into HIV-1 persistence in vivo.

### 6.3. Simultaneous Integration Site Profiling and Proviral Sequencing Captures HIV-1 Clonal Expansion Dynamics of Intact versus Defective Proviruses

In 2019, two groups [64,65] developed novel methods to capture the HIV-1 integration site and near-full length HIV-1 proviral genome of the same provirus: the matched integration site and proviral sequencing (MIP-seq) by the Lichterfeld group [65] and whole genome amplification-single genome sequencing (WGA-SGS) by the Kearney and Coffin groups [64]. These methods allowed for mapping of the clonal expansion dynamics of intact and defective HIV-1 proviruses, and interrogation of the impact of HIV-1 integration into cancer-related genes on HIV-1 persistence. Both groups used multiple displacement amplification (MDA, also called whole genome amplification) mediated by the phage phi29 polymerase to amplify DNA to multiple copies and then separated the amplified DNA into two aliquots for integration site and near-full length HIV-1 proviral genome sequencing, respectively. Briefly, DNA from clinical samples is plated at limiting dilution (each well contains only one HIV-1-infected cell out of many uninfected cells). The bulk DNA in each well is universally amplified by phi29 polymerase, resulting in many copies of the genome in the well. The amplified DNA is then separated for near-full length HIV-1 proviral genome sequencing and integration site analysis [65]. This method has recently been expanded to capture HIV-1 RNA in addition to HIV-1 integration site and proviral DNA genome by the Lichterfeld group [66], and termed Parallel HIV RNA, Integration Site and Proviral Sequencing (PRIP-Seq). Briefly, cells from HIV-1-infected individuals are plated at limiting dilution (such as thousands of cells per well, but no more than one HIV-1-infected cell per well, based on Poisson distribution). Then, cellular DNA and RNA are extracted into different aliquots. The cellular DNA aliquots are used for MIP-seq to capture HIV-1 integration site and near full-length proviral genome, while the matched cellular RNA aliquots are used for targeted HIV-1 RNA amplification. PRIP-seq allows understanding not only HIV-1 clonal expansion dynamics but also whether HIV-1 integration site affects HIV-1 expression levels. By mapping HIV-1 integration sites to chromatin accessibility data obtained from separate aliquots of cells, Einkauf et al. found that that actively transcribed genes have open chromatin accessibility and activating chromatin marks, and less transcribed genes have closed chromatin accessibility and repressive chromatin marks, consistent with normal human gene expression control [66]. HIV-1 proviruses that are integrated in highly transcribed genes, some of which are highly expanded clones, have higher HIV-1 RNA expression levels, and HIV-1 proviruses that are integrated into less transcribed genes have lower HIV-1 RNA expression level [66]. These results show that HIV-1 expression level is impacted by local human gene expression control at the integration site.

### 6.4. The Enrichment of HIV-1 Integration into Heterochromatin Reflects Immune Selection Pressure against Actively Transcribed HIV-1

Using MIP-seq to profile HIV-1 integration site and proviral landscape in specific clinical cohorts of interest, such as a group of elite controllers, Jiang et al. found that clonally expanded HIV-1 proviruses in elite controllers are located in transcriptionally inactive sites with lower chromatin accessibility, with some even in heterochromatin regions such as the centromere [67]. Interestingly, the Bangham group has observed a similar pattern for HTLV-1 in 2011: they noted that the immune negative selection dominates during chronic HTLV-1 infection, favoring the survival of proviruses integrated in transcriptionally silenced DNA, particularly in asymptomatic HTLV-1 carriers [61]. Given that elite controllers have strong CTL responses, the enrichment of intact HIV-1 proviruses in heterochromatin regions in elite controllers is not caused by preferential integration into these regions or specific silencing mechanisms in these individuals; instead, this is a result of strong CTL pressure that eliminated cells harboring HIV-1 proviruses not integrated into heterochromatin. Cells harboring intact HIV-1 proviruses that are integrated into transcriptionally unfavorable heterochromatin locations are the survivors under the strong CTL responses. Cells harboring intact HIV-1 proviruses that are integrated into open chromatin regions were eliminated by CTLs during the course of long-term infection [67]. Nevertheless, intact HIV-1 proviruses that are integrated into heterochromatin are extreme and rare cases; elite controllers may have clonally expanded replication-competent HIV-1 [68] and HIV-1 integration into actively transcribed genes, as reported previously by the Blankson group [69].

## 7. HIV-1 Insertional Mutagenesis: The Location-and Orientation-Dependent Impact of HIV-1 Integration into Cancer-Related Genes on HIV-1 Persistence

Multiple studies have identified the enrichment of HIV-1 integration sites in various genes, but some genes, such as *BACH2*, have been independently and repeatedly identified in different cohorts by a wide variety of methods [23,53,63,70,71,72]. Similar to the heterogeneity of oncogenic mutations in cancer, HIV-1 insertional mutagenesis provides survival benefit to the infected cells depending not only on whether the gene is a cancer-related gene or not, but also how HIV-1 integration affects the function of the gene. HIV-1 integration that leads to the gain of function of an oncogene or the loss of function of a tumor suppressor gene provides the survival benefit to the infected cell, and proliferation after prolonged infection. Therefore, this effect is location- and orientation-dependent: the impact of HIV-1 integration site on HIV-1 persistence requires integration into a 5′ intron and in the same orientation as the transcription unit [23,39,63] (Figure 2).

The question then becomes: is this enrichment in certain cancer-related genes caused by preferential integration during acute infection or by preferential persistence and expansion after long-term infection? HIV-1 integration sites are enriched in the introns of actively transcribed genes [59,70]. Although HIV-1 integration can be found in all chromosomes, there is a preferential enrichment in chromosomes 16, 17, and 19 both in HIV-1-infected individuals [70,73], cell line models [74] and animal models [75], since these chromosomes have high gene density [70,73]. During acute infections HIV-1 integration into certain cancer-related genes such as *BACH2* is observed; however, it is not enriched. Therefore, rather than preferential integration into these genes during acute infection, the enrichment into certain cancer-related genes such as *BACH2* is more likely caused by the survival benefit and preferential proliferation of these cells after long-term infection.

### 7.1. Lessons from Acute versus Chronic Infections: Clonal Expansion of HIV-1-Infected Cells Established during Acute Infection Persists after Viral Suppression

Given that more than 50% of the HIV-1 latent reservoir undergoes clonal expansion [20,21,22], it is critical to study when these clonally expanded HIV-1-infected cells were first established. By analyzing HIV-1 proviral sequences before and after ART, Coffin et al. identified clonally expanded cells during acute infection (as early as four weeks after infection) and persisted for years after viral suppression [76]. Separately, von Stockenstrom et al. found that the HIV-1 sequences are highly diverse before ART and highly clonal after suppressive ART [77]. In some cases, the HIV-1 proviruses in the clonally expanded HIV-1-infected cells after long-term ART are found to be phylogenetically identical to the HIV-1 plasma sequences before ART [77]. These results suggest that during acute infection, HIV-1-infected cells are highly diverse and are not clonal. Furthermore, HIV-1 integration into cancer-related genes such as *BACH2* and *MKL2* was identified during acute infection. During untreated infection, the ongoing battle between the host immune selection pressure and viral replication and mutation continues to change the HIV-1 proviral landscape. However, upon initiation of ART, new rounds of infection are blocked. Infected cells that survived immune selection pressure before ART [78] or had survival benefits by integrating into cancer-related genes [23,63] gradually dominate the HIV-1 latent reservoir, undergo clonal expansion, and persist over time. Of note, in comparing the polyclonal nature of HIV-1-infected cells in acute infection with the oligoclonality seen during treatment with ART, it is essential to ensure that the sample depth is adequate in the sequencing in each case. The danger is that oligoclonal proliferation during acute infection might be missed because the number of infected cells (of diverse clones) is so large that repeated detection of an expanded clone is unlikely. More studies are needed to compare the clonal expansion dynamics during viremia and after viral suppression.

### 7.2. Lessons from Pediatric versus Adult Infections: Clonal Expansion of HIV-1-Infected Cells in Children Is Comparable to That in Adults

Despite discrepancies between the pediatric and adult immune landscapes–such as the higher proportion of memory cells, the gradual decline of T cell repertoire diversity, and the level of immune aging in adults–little evidence suggests that the HIV-1 latent reservoir is significantly different in children. Katusiime et al. used near full-length proviral amplification and sequencing (NFL-PAS) to analyze proviral sequences extracted from peripheral blood mononuclear cells (PBMCs) of eight children who had been treated with ART for 6–9 years. Seven of 733 sequences identified were intact; these seven provirus sequences came from children who had initiated ART after 2.3 months of age, proving that intact proviruses can be identified in children treated with ART [79]. Furthermore, two identical proviral sequences were discovered in one individual, suggesting clonal expansion (although no integration site analysis was performed) [79]. In a separate study, Bale et al. examined the PBMCs of 11 children who initiated ART between 1.8 and 17.4 months of age [80]. Using integration site analysis on samples before and during ART samples, this study identified evidence of clonal expansion in 10 out of 11 children. In 8 out of 11 children, clones identified pre-ART persisted throughout the 6–9 years of ART, suggesting that clonally expanded cells are established before ART [80]. The HIV-1 integration site patterns are similar to those observed in adults [80], including enrichment of integration into cancer-related genes *BACH2* and *STAT5B* with the same orientation to the transcription units [23,63]. Thus, as in adults, the latent reservoir is established early in children and may be maintained via HIV-1-integration site-dependent proliferation, similar to infections in adults.

### 7.3. Lessons from Primary Cell Models Support the Location-and Orientation-Dependent Impact of HIV-1 Integration into Cancer-Related Genes on HIV-1 Persistence

By examining HIV-1 integration site datasets, the Hughes and Coffin group found that six genes (*BACH2, STAT5B, MKL2, IL2RB, POU2F1,* and *MYB)* are enriched in the HIV-1-integration sites in clonally expanded HIV-1-infected cells [81]. Indeed, HIV-1 integration into these genes, particularly *BACH2*, *STAT5B*, and *MKL2*, are repeatedly seen in multiple studies from different cohorts using different methods [23,63,72]. In particular, HIV-1 integration into these genes shares the same characteristics of location and orientation dependent enrichment (Figure 2): (a) enrichment of integration sites in 5′ introns, and (b) a preference for the proviruses to integrate in the same orientation as the host gene’s transcription units. Yet, HIV-1 integration into genes other than these six can also contribute to clonal expansion. For example, HIV-1 integration into *STAT3*, a gene that has been linked to uncontrolled cell growth and oncogenesis, is found to be enriched in primary cell models [82]. In this primary cell model, HIV-1 integration into *STAT3* is also enriched in a location and orientation dependent manner—there is an enrichment of HIV-1 integration in the same orientation as *STAT3* and in the 5′ introns (upstream of exon 2). In another example, HIV-1 integration into an oncogene *VAV1*, which has been identified in virally suppressed individuals [63], creates HIV-1-driven aberrant VAV1 protein expression, obliterates the N-terminal regulatory region of this oncogene, and provides survival benefit to the infected cells [39]. Therefore, HIV-1 integration sites that are enriched in long-term in vitro culture support the findings on how HIV-1-infected cells persist by having proviruses integrate in cancer-related genes in a location- and orientation-dependent manner, although the exact HIV-1 integration site may be different from those observed in vivo (i.e., *STAT3* identified in vitro [82] is not one of the six enriched genes identified in vivo [81]).

### 7.4. Lessons from Animal Models Support the Location-and Orientation-Dependent Impact of HIV-1 Integration into Cancer-Related Genes on HIV-1 Persistence

Studying HIV-1 integration events in animal models is an alternative method of tracking HIV-1 integration sites in vivo. Two studies examined HIV-1 integration in humanized mouse models. Briefly, immunodeficient mice (such as nonobese diabetic–SCID common γ^–/–^ (NSG) neonatal mice) were engrafted with human fetal liver CD34^+^ hematopoietic stem and progenitor cells to establish human CD4^+^ T cells in mice that allowed for HIV-1 infection in the peripheral blood and tissues [75,83]. HIV-1 integration sites in humanized mouse models after 12 weeks of HIV-1 infection seem to be similar to those observed in human infections, with an enrichment of HIV-1 integration in the gene-dense chromosomes 16, 17, and 19. In these animal models without ART treatment, Haworth et al. found enrichment of HIV-1 integration into *JAK2* and *SEPT9* in clonally expanded cells [75], indicating that clonal expansion occurs during acute infection. Pathway analysis of HIV-1 integration sites in clonally expanded cells are enriched in viral process-related genes [75,83]. This is potentially because viral process-related genes during acute HIV-1 infection are actively transcribed, have open chromatin, and have a higher chance for HIV-1 pre-integration complex to access. When comparing HIV-1 integration sites during early (2 weeks) versus late (15 weeks) infection, they found enrichment of integration into genes that are involved in cellular proliferation at later time points [83]. The strength of HIV-1-infected humanized mouse models is the ability to capture early infection events and profile HIV-1 infection at tissue sites, which is typically hard in human studies. The weakness of humanized mouse models is that it is difficult to recapitulate long-term viral suppression (>6 months of undetectable viral load) given the short lifespan of mice (~1 year). Nonetheless, HIV-1-infected humanized mouse models can recapitulate the enrichment of HIV-1 integration into cancer-related genes in a location- and orientation-dependent manner, although the exact HIV-1 integration sites may be different from those observed in vivo.

## 8. HIV-1 Insertional Mutagenesis Can Cause Aberrant HIV-1-Driven Proliferation That is out of Control from the Host

The dogma-shifting discovery of clonal expansion of HIV-1-infected cells harboring HIV-1 integration into specific sites of certain cancer-related genes [23,63] raises questions that are mechanistically intriguing and clinically important: what does HIV-1 do at the integration site and why is it important? HIV-1 integration into host introns is known to cause transcriptional interference on the host gene through promoter occlusion or read-through transcription from the 3′ LTR [84,85]. Using PCR primers targeting the HIV-1 and the two HIV-1 integration enriched genes *BACH2* and *STAT5B*, Cesana et al. found HIV-1 RNA splicing into the RNA of *BACH2* and *STAT5B*, creating HIV-1-*BACH2* and HIV-1-*STAT5B* chimeric RNA, respectively [72]. These findings suggest that HIV-1 integration and aberrant splicing alters the host transcriptional landscape of the genes into which HIV-1 is integrated.

### 8.1. HIV-1 Integration Changes the Host Gene Transcriptional Landscape at the Integration Site through Aberrant Splicing and Read-through Transcription

To examine HIV-1-host interactions beyond the known enrichment in *BACH2* and *STAT5B*, our group developed HIV-1 SortSeq to capture HIV-1 RNA^+^ cells from virally suppressed individuals [39]. HIV-1 SortSeq uses HIV-1 RNA expression as a surrogate to capture HIV-1-infected cells using fluorescent in situ hybridization (FISH)-based HIV-1 RNA staining and flow cytometric sorting to capture HIV-1-infected cells for total RNA sequencing. As opposed to high-throughput droplet-based or microwell-based platforms, which only capture short (50–100 bp) RNA near the 3′ poly-A tail (in cases of polyT-based capture) or 5′ transcripts (in cases of template switching by Moloney murine leukemia virus reverse transcriptase), total RNA sequencing (after ribosomal RNA removal) of 2 × 150 bp RNA fragments allows us to capture the junction between HIV-1 and host RNA genome. Therefore, in addition to capturing HIV-to-host RNA splicing between HIV-1 and *BACH2*, we captured different types of aberrant splicing between HIV-1 and the host RNA in CD4^+^ T cells from virally suppressed, HIV-1-infected individuals: (a) HIV-1-driven read-through transcription from the 3′ LTR into host RNA in the same orientation (*MTOR*, *KANSL3*, *TTN*, *NUB1*, and *NSFL1C*) or convergent orientation (*SIK3*, *STARD9*, *FBXL5*, *DPYD*, and *UMAD1*), (b) host-driven read-through transcription from host intron into HIV-1 5′ LTR (*NBPF3*), (c) HIV-1-driven splicing from HIV-1 major splice donor (MSD) into the host exons (*BACH2* and *NFATC3*), and (d) host-driven splicing from host exon into HIV-1 acceptors A4a (from *MIR155HG*) or A5 (from *SMARCC1* and *PYHIN1*). This HIV-1-to-host RNA splicing event was also found in primary cell models (*STAT3*) [82] and cell line models (*VAV1*, *RAP1B*, and *SPECC1*) [39]. Our findings indicate HIV-1 integration can change the host gene transcription landscape at the integration site by readthrough transcription and splicing, both from HIV-1 to host gene RNA and from host gene to HIV-1.

### 8.2. HIV-1 Promoter Dominates over the Host Promoter and Drives High Levels of Cancer Gene Expression at the Integration Site

HIV-1 integration changes the transcriptional landscape beyond readthrough transcription or splicing. Using RNA sequencing (as opposed to targeted PCR), we found that HIV-1 LTR promoter dominates over the host promoter and determines the expression level of the host gene in which HIV-1 is integrated. Using CRISPR-mediated activation and inhibition of HIV-1 LTR, we found that activation of HIV-1 LTR induces higher levels of HIV-1-driven aberrant cancer gene expression at the integration site, whereas inhibition of HIV-1 LTR suppresses HIV-1-driven aberrant cancer gene expression [39] (Figure 2). Drugs that induce HIV-1 gene expression (such as PMA/ionomycin) increase HIV-1-driven aberrant cancer gene expression, while drugs that suppress HIV-1 expression (such as JAK inhibitor filgotinib) also suppress HIV-1-driven aberrant cancer gene expression [86]. Taken together, HIV-1 drives aberrant and high levels of host gene expression during HIV-1 reactivation. When HIV-1 is integrated in specific locations of cancer-related genes, HIV-1 drives aberrant proliferation and provides survival benefit to the cells during long-term infection.

### 8.3. HIV-1 Insertional Mutagenesis May Cause Cancer Transformation in the Infected Cells in Rare Cases

HTLV-1 causes adult T cell leukemia/lymphoma (ATL) in the infected cells. However, although HTLV-1 is well-known to be oncogenic, it takes more than 50 years for HTLV-1 to induce cancer of the infected cells in around 5% of infected individuals [87]. HIV-1 infection does not cause cancer in the infected cells themselves, except for one case report [88]. In this unique case, a defective HIV-1 provirus integrated upstream of the first exon of *STAT3*. The HIV-1 3′ LTR promoter drives high levels of *STAT3* expression, which is associated with cellular proliferation [88]. To understand whether HIV-1 insertional mutagenesis would cause cancer in HIV-1-infected individuals, Hughes et al. took cutaneous T cell lymphoma tissues and examined HIV-1 integration sites in these cancers [89]. Strikingly, they found HIV-1 integration immediately upstream of the first intron of *STAT3* (in five cancer tissues from three participants) and *LCK* (in three tissues from two participants), in the same transcriptional orientation as the host gene [89]. These HIV-1 proviruses were defective, with a large internal deletion in *gag* and *pol.* We have previously showed that defective HIV-1 proviruses can have functional HIV-1 promoter, can be readily transcribed and translated, and can activate cryptic splice sites for RNA splicing if canonical splice sites are deleted [19]. Indeed, the intact HIV-1 3′ LTR of these defective proviruses drives the expression of HIV-1-*STAT3* chimeric RNA [89]. Aberrant RNA splicing from upstream host RNA into HIV-1 *tat* allows Tat expression and efficient HIV-1 expression [89]. The 3′ LTR driven HIV-1-*STAT3* chimeric RNA was expressed at a rate thirty times greater than endogenous *STAT3* RNA driven by the *STAT3* promoter [89]. Overall, these striking findings suggest that HIV-1 insertional mutagenesis, HIV-1-driven cancer-related gene expression, and aberrant RNA splicing between HIV-1 and host gene not only serve as mechanisms for clonal expansion and HIV-1 persistence, but also induce cancer formation in the infected cells in rare cases.

### 8.4. Why Does HIV-1 Infection Cause Cancer in the Infected Cells Only in Extremely Rare Cases?

It remains unclear why HIV-1 causes cancer in the infected cells only in very rare cases, compared with a much higher frequency of malignant transformation in HTLV-1-infected cells. First, HTLV-1 can cause cancer by several mechanisms [87]. For example, HTLV-1 proteins Tax and HBZ promote cellular proliferation through canonical and non-canonical NF-κB activation [90]. HTLV-1 *HBZ* mRNA increases cellular proliferation through transcription factor E2F1 expression [91]. HTLV-1 alters host gene expression by changing the CTCF-mediated chromatin looping landscape through the CTCF binding site within the HTLV-1 genome [92]. In contrast, HIV-1 viral proteins are not known to cause cancer. HIV-1 Tat may induce NF-κB activation [93], but HIV-1 Tat expression is fluctuating [94] (as opposed to persistent *HBZ* expression in HTLV-1 infection [91]). HIV-1-driven aberrant cancer gene expression is the only known mechanism that may induce malignant transformation. Given that malignant transformation requires multi-hits [95], HIV-1 integration into cancer genes is not sufficient to cause cancer. Other pre-existing cancer mutations in the infected cells are likely required to transform the infected cells into cancer. Since HIV-1 only integrates into one allele in the chromosome, HIV-1 integration into cancer genes can induce malignant transformation only if this mutation is dominant, if the gene is haplo-insufficient, or if the other allele has intrinsic inactivating mutations. Finally, even for HTLV-1 infection, it takes more than 50 years for a small proportion (5%) of infected individuals to develop cancer [87]. Since humans acquired HIV-1 infection relatively recently [96], with the first cases identified around 40 years ago in the 1980s [97], it may take more time to know the incidence of HIV-1-induced cancer in infected cells in infected individuals.

## 9. Conclusions

Uncontrolled gene expression is a hallmark of cancer. When HIV-1 integrates into specific locations of cancer-related genes and causes a gain of function in oncogenes or a loss of function in tumor suppressor genes, HIV-1 can drive aberrant proliferation of the infected cells. Unlike antigen-driven proliferation and homeostatic proliferation, which are under host control, HIV-1 insertional mutagenesis may induce uncontrolled proliferation of the infected cells and eventually–after decades of infection in rare cases–cause cancer transformation of the infected cells. While ART effectively suppresses new rounds of infection, the HIV-1 LTR in the existing infected cells remains active and functional. HIV-1 LTR promoter continues to drive the proliferation of infected cells despite long-term suppressive ART. Drugs that can suppress HIV-1 LTR promoter activity, which are not yet available, should be considered as a therapeutic strategy in addition to current ART to halt the proliferation of HIV-1-infected cells.

## Data Availability

Not applicable.
